# Tenabine

**Published:** 1886-05

**Authors:** 


### TEREBENE.

This substance has lately been employed by Dr. William Murrell, of London, in the treatment of winter cough, and he published a series of one hundred and fourteen cases in which great benefit had been derived from its use. Expectoration is facilitated and the shortness of breath is relieved. It is a colorless fluid, with a faint terebinthinate odor, which is by no means disagreeable. The remedy may be inhaled as a vapor with the ordinary "respirator," or it may be atomized by means of one of the pulverizateurs, several forms of which are in use, and the fine spray so produced inspired by the patient. For internal administration by the mouth, it may be given as an emulsion with gum tragacanth. Another, and possibly a preferable manner, is to give it dropped on a lump of sugar, and this method has the advantage of being small in bulk and easily carried about by the patient, so as to allow of its being repeated as often as may be thought advisable. It is best to begin with five or six drops on sugar every four hours and gradually to increase this dose to twenty minims. This is, for most people, the maximum quantity, but the drug, Dr. Murrell says, has little or no toxic action, and considerably larger doses have been taken without the production of any untoward symptoms.

Chemically terebene $C_{10}H_{16}$, is derived from oil of turpentine, either by the action of one part of concentrated sulphuric acid on twenty of the oil and subsequent distillation, or by one of the other processes known to chemists. When terebene is submitted to the action of dry hydrochloric acid gas, a white crystalline mass results, the hydrochlorate of terebene, commonly known as turpentine camphor, or terpine, which resembles ordinary camphor, and has also been employed in the treatment of the same class of complaints as terebene.

Some difficulty was experienced in obtaining an adequate supply of the pure drug when first brought forward, and attempts were made to pass off one or more patent preparations on the public as the only pure terebene, but it is to be hoped without much success. —*Medical Bulletin.*

**The** Arch Duke, Charles Theodore of Bavaria, who is a thoroughly educated physician and a practitioner, has become a member of Pasteur's corps of assistants in Paris.

